# Spatiotemporal analysis of nontraditional security issues evolution globally: evidence from news big data

**DOI:** 10.1038/s41598-026-42600-1

**Published:** 2026-03-11

**Authors:** Jiaqi Li, Zhenfu Li, Shiyue Li, Qiqi Zhang, Yutao Zhou, Meng Sun

**Affiliations:** 1 College of Transportation Engineering, Dalian Maritime University, Dalian, 116026 China; 2 College of Shipping Economy and Management, Dalian Maritime University, Dalian, 116026 China; 3 Federation of Industry and Commerce of Honggang District, Daqing, 163511 China; 4 School of Geography and Tourism, Qilu Normal University, Jinan, 250222 China; 5 College of Business, Shandong Normal University, Jinan, 250014 China

**Keywords:** Nontraditional security (NTS), Event data analysis, News big data, GIS visualization, Security hotspots, Sustainability, Social evolution

## Abstract

Since the twenty-first century, global non-traditional security (NTS) challenges have increasingly threatened society. This study uses event data from the Global Database of Events, Language, and Tone (GDELT) to examine how the frequency and impact of NTS issues have evolved worldwide from 2000 to 2023. Building on a newly constructed global panel dataset covering 153 countries, two complementary indicators, the CEN (count the number of event) and NSII (non-traditional security impact index), are calculated to measure the scale and severity of NTS activities. The analysis identifies five distinct phases in the global evolution of NTS issues, with peaks in 2012 and 2016 driven primarily by surges in terrorism and social instability in the Middle East, South Asia, and parts of Africa. Over time, NTS hotspots have gradually shifted toward more dispersed and transregional patterns, reflecting the diffusion of risks across interconnected systems. These findings underscore the need for adaptive, cross-border governance and continuous monitoring mechanisms. This study contributes a global, data-driven framework for tracking the spatiotemporal evolution of NTS risks and informing policy responses.

**Supplementary Information:**

The online version contains supplementary material available at 10.1038/s41598-026-42600-1.

## Introduction

Non-traditional security (NTS) has gradually evolved from a conceptual extension of national security to a multidimensional framework encompassing political, economic, environmental, societal, and human dimensions. Earlier theoretical paradigms including the Copenhagen School’s securitization theory^1^, critical approaches^2^, and human security perspectives^3^—collectively emphasized a shift from state-centric to human-centric understandings of security. While these frameworks have enriched the conceptual foundation of NTS, extensive theoretical debates alone have provided limited empirical insight into how NTS challenges manifest, evolve, and interact at the global level.

Since the 9·11 terrorist attacks with the unprecedented global impact in 2001, attention on NTS threats is gradually increased ranging from terrorism and pandemics to climate-related disasters, transnational crime, and cyber insecurity^4,5^. However, existing quantitative research remains fragmented. Many studies focus narrowly on single issues (e.g., terrorism or health crises) or specific regions. Few have developed integrated frameworks capable of tracing how multiple categories of NTS events co-evolve and spatially cluster over time.

To address this gap, this study constructs a global panel dataset of NTS events from 2000–2023, covering 153 countries and introduces two composite indices CEN (count of event frequency) and NSII (non-traditional security impact index) to measure the magnitude and intensity of NTS dynamics. Using spatiotemporal analysis, the study aims to provide a systematic account of how NTS challenges evolve and concentrate across regions and periods. This design provides a transparent, replicable account of how NTS frequency and impact co-evolve and where durable hotspots emerge^6^. This design choices follow recent guidance on event-data curation and comparability, emphasizing clear inclusion criteria, harmonized variable definitions, and replicable aggregation rules. Consistent with recent methodological work on spatial analysis, we rely on map-based diagnostics and scale-aware procedures to summarize spatial patterns, acknowledging the sensitivity of spatial dependence to specification choices^7,8^. These indices operationalize NTS complexity into comparable, quantitative indicators that allow for the examination of spatial and temporal evolution. This approach represents a methodological innovation over prior single NTS issue research, which typically examined isolated threats.

Nevertheless, relying on media-derived event data such as GDELT introduces inherent methodological challenges. Coverage and salience often reflect media-system characteristics—including Western-centric reporting, censorship, language accessibility, and a tendency to emphasize conflictual. These biases may affect cross-country comparability. Recognizing these constraints, this study applies normalization and validation procedures to mitigate bias, while acknowledging that its results capture the global media footprint of NTS activity—an important dimension of perceived security that influences international policy attention.

In summary, this study aims to bridge theoretical and empirical gaps in global NTS research by developing a reproducible, data-driven framework to monitor and interpret long-term changes in NTS dynamics. Specifically, it seeks to answer three research questions:

RQ1: How have global NTS frequency (CEN) and impact (NSII) co-evolved over time?
RQ2: Where are the geographical hotspots of NTS events, and how have their spatial distributions shifted over time?
RQ3: What contextual factors help explain the regime shifts in the global evolution of NTS issues?

This work contributes to quantitative security studies by integrating big event data and GIS-based visualization into the analysis of global NTS issues, providing empirical evidence for policy design and cooperative governance in an increasingly interconnected world.

## Literature review

The concept of non-traditional security (NTS) has evolved through successive theoretical refinements since the late twentieth century. Early explorations by Ullman^9^ and Dziedzic^10^ extended the notion of security beyond military threats to encompass poverty, disease, environmental degradation, and transnational crime, while Buzan and Smith emphasized the human and societal dimensions of security rather than state-centric protection^11,12^. Collectively, these works laid the conceptual foundation for viewing NTS as a multidimensional framework encompassing political, economic, environmental, and human concerns. With economic development, technological progress, and deepening globalization, NTS studies have increasingly focused on emerging global issues such as terrorism, climate change, infectious diseases, and cybersecurity.

Following the end of the Cold War and the decline of conventional military threats, global non-military challenges—including drug trafficking, illegal migration, and ecological degradation—became more salient^13,14^. Since the September 11 terrorist attacks, NTS research has accelerated dramatically^4,15^, evolving from conceptual debate to theoretical consolidation^16^. Within this evolution, several theoretical schools have contributed distinct perspectives, such as the Copenhagen School’s securitization theory, Critical Security Studies, Feminist and Human Security approaches, and China’s “harmony”-based framework^1–3,17–22^. Together, these perspectives have broadened the analytical scope of security studies and established NTS as an integral field of contemporary international relations.

Empirical NTS research has likewise diversified thematically and geographically. First, specific NTS have been major focuses. Terrorism^23^ and border disputes^24^ have received substantial scholarly attention over an extended period, while water security^25^, food security^26^, and energy security^27^ have been extensively studied due to their fundamental roles in sustainable development. In recent years, scholars have also turned their focus to cyber security^28^, cultural security^29^, ecological security^30^, and public security^31^. Second, geographically, research has primarily concentrated on specific regions such as China’s western borderlands^32^, Central Asia^33^, East Africa^34^, Latin America^35^, and Europe^36^. However, global-scale quantitative analyses integrating multiple NTS domains remain rare. Most existing work emphasizes theoretical exposition or single-issue empirical models, leaving limited understanding of how diverse NTS threats interact across regions and time^6^.

Given these limitations, scholars have increasingly called for data-driven and globally comparable approaches to NTS measurement. Traditional datasets drawn from meteorology, resource management, or terrorism research are often fragmented and inconsistent in structure, limiting cross-domain analysis. Recent advances in large-scale event data offer a way forward. News-based datasets—characterized by real-time coverage, accessibility, and global reach—enable systematic analysis of NTS patterns^37^. Among these, the GDELT database^38^ provides the most comprehensive source, tracking events in over 100 languages and supporting applications in geopolitical and international security research^39,40^. In adapting such datasets, researchers must remain attentive to selection and reporting biases consistent with best practices^41^. The present study adopts transparent sourcing and normalization procedures to ensure comparability and validity in measuring NTS dynamics.

## Data

The dataset of NTS events primarily originates from the GDELT Event Database. The GDELT project utilizes its Translingual platform for real-time streaming English translation, gathering global news since 1969, and updating every 15 min. In applied research using GDELT project data, the most frequently utilized databases are the Event Database and the Global Knowledge Graph (GKG). The Event database used in this study spans from January 1, 1979 to now, and includes 58 fields such as event country, event type, occurrence time, average tone, article mentions, and Goldstein scale, among others. It covers over 20 major event types (comprising over 300 sub-types) classified into substantive cooperation, verbal cooperation, substantive conflict, and verbal conflict. This database integrates participating countries in events, identified as Actor1Country and Actor2Country. And variables can be retrieved and processed using SQL language on the Google Cloud platform through Google BigQuery.

In addition to GDELT, several other well-known event datasets have been widely used in security and conflict studies, such as the Armed Conflict Location and Event Data Project (ACLED)^42^, the Integrated Crisis Early Warning System (ICEWS)^43^, and the Uppsala Conflict Data Program (UCDP)^44^. These datasets provide high-quality, manually curated information on political violence, protests, and armed conflicts. However, their thematic coverage is relatively narrow and primarily focused on state-based or conflict-related events. By contrast, GDELT offers a broader and more continuous record of non-traditional security (NTS) activities, including health crises, cyber incidents, and humanitarian emergencies. This multidimensional scope makes GDELT particularly suitable for constructing composite indicators that reflect the evolving global landscape of NTS issues.

Although GDELT is the most comprehensive global news event database, it remains affected by structural biases in media representation. Western and English-language sources tend to dominate, while countries with limited press freedom may be underrepresented. These potential biases were acknowledged and mitigated through normalization and aggregation procedures.

From the GDELT Event Database, we extracted all events between January 1, 2000 and December 31, 2023, selecting variables “Year”, “Actor1CountryCode”, “Actor2CountryCode”, “QuadClass”, and “Eventcode”. Existing variables like “GlobalEventID”, “GoldsteinScale”, “AverageTone”, “NumMentions”, and “NumArticles” were aggregated or averaged to generate new metrics including CEN, AGS, AAT, SNMT, and SNA (detailed definitions in Table 1). Goldstein scores and sentiment (AAT) serve as proxies for conflict–cooperation intensity and public tone, respectively—two well-established dimensions in quantitative security and political communication research. While we recognize that these proxies are imperfect, they provide a standardized, reproducible way to capture the perceived impact of NTS events in global media.

**Table1:** Definition of selected variables.

Variable name(datatype)	Variable source	Variable defination
Actor1CountryCode (string)	Extracted directly from the GDELT database	This field records the three-character country code corresponding to Actor1’s national affiliation in the event. It may remain empty if Actor1 cannot be recognized or its country cannot be ascertained by the system
Actor2CountryCode (string)	Extracted directly from the GDELT database	This field denotes the country associated with Actor2, following the same format and definition as Actor1CountryCode
Quadclass (integer)	Retrieved from GDELT as a classification of event type	Events are classified into four categories based on this numeric field, known as Quad Classes: 1 = Verbal Cooperation, 2 = Material Cooperation, 3 = Verbal Conflict, 4 = Material Conflict
Eventcode (string)	Taken from GDELT; represents the CAMEO action code	This field records the unprocessed CAMEO action code representing the activity carried out by Actor1country in relation to Actor2country
CEN (integer)	Computed by counting the number of distinct GlobalEventID entries in GDELT	GlobalEventID uniquely identifies each event in the dataset, while CEN reflects the frequency or count of such events
AGS (floating point)	Calculated as the average of the GoldsteinScale values from GDELT	The Goldstein Scale assigns each CAMEO event code a value between -10 and + 10 based on its expected impact on national stability. AGS captures the mean of these values across events
AAT (numeric)	Derived by averaging the Average Tone scores associated with the events in GDELT	Average Tone measures the average sentiment of documents reporting an event during its initial 15-min detection window, on a scale from -100 (very negative) to + 100 (very positive), with typical values clustering near neutral (0). AAT denotes the average of these Average Tone values across events
SNMT (integer)	Obtained by summing the NumMentions values from GDELT	NumMentions records how many times an event is mentioned in all documents within the 15-min window of its first detection, including repeated mentions in the same source. As a measure of event salience, SNMT captures the total sum of these mentions across events
SNA (integer)	Obtained by summing the NumArticles values from GDELT	NumArticles measures how many unique documents mention an event during its initial 15-min observation window. It serves as an indicator of the event’s prominence. SNA is the cumulative sum of these counts across events

The selection of 79 CAMEO codes was based on their relevance to non-military and cross-border risks (e.g., humanitarian crises, protests, environmental events, cyber incidents). The complete code list and rationale are provided in Table A1 of the supplementary material.

The final dataset forms an unbalanced country–year panel of 153 countries over 24 years, covering all major global regions (Regional Distribution of Sample Countries in Table 2). Countries with missing or zero entries were retained to reflect reporting variation rather than data absence. Imputation was not performed. These structured data serve as the foundation for constructing the Nontraditional Security Impact Index (NSII) and conducting subsequent spatial and temporal analyses.

**Table 2:** Regional distribution of sample countries.

Region	The number of countries
East Asia and the Pacific Region	25
Latin America and the Caribbean Region	22
South Asia	6
Europe and Central Asia	35
Sub-Saharan Africa	46
Middle East and North Africa	19
Total	153

Given the use of automated global media data, this study follows ethical data-use principles, avoiding individual identification and recognizing that news coverage may not equate to real-world severity. Results are interpreted as reflecting perceived rather than absolute security conditions.

## Construction of nontraditional security impact index (NSII)

### Data processing

Although the concept of non-traditional security (NTS) has been widely discussed in the existing literature, there is no clear criterion for distinguishing NTS-related issues from those that are not. The only universally accepted definition of NTS is that it is characterized by non-military feature^45^. Therefore, the NTS events selected for this study must satisfy two conditions: first, they must exhibit non-military characteristics; and second, they must pose a threat to the security of individuals, society, or the state.

To ensure the accuracy and relevance of non-traditional security (NTS) event extraction from the GDELT Event Database, a multistep filtering and aggregation process was conducted.

First, only events with clear country identifiers for both actors (i.e., Actor1CountryCode and Actor2CountryCode) were retained. We recognize that this conservative choice may under-represent domestic or under-coded events (e.g., single-actor reports). This conservative rule reduces misattribution but can exclude domestic or under-coded events. As a sensitivity check, we also aggregated using a relaxed rule that accepts records with at least one actor country code (preferring Actor1CountryCode, otherwise ActionGeo_CountryCode when available). We therefore retain the conservative baseline and note the potential selection effect. Second, a selection of 79 event codes was made based on the CAME^46^ classification (see Table A1), ensuring that retained events possess non-military characteristics and pose potential threats to individual, societal, or national security. This ensures alignment with the universally accepted definition of NTS as “non-military threats to security”. Third, five key attributes were extracted and aggregated to characterize each country’s annual NTS status: CEN (Number of distinct NTS events); Goldstein Scale and Average Tone (Reflecting conflict/cooperation severity and media sentiment respectively); SNMT and SNA (Capturing media attention and breadth of coverage).

To reduce potential distortions from outlier events or media overexposure, logarithmic transformation and normalization were applied where appropriate. Averaging across Goldstein and tone values captures general tendencies rather than individual outliers.

The year 2000 was chosen as the starting point due to the limited reliability of GDELT data prior to the widespread digitalization of global media coverage after the late 1990s. Country codes were standardized using the ISO three-letter format. After cleaning missing or ambiguous entries, a final total of 1,070,966 valid event records was compiled for index construction and spatial analysis.

The sources and mitigation of media and reporting bias. Media-derived event data can vary systematically across countries and over time due to (i) cross-lingual coverage differences, (ii) censorship and uneven access to information, and (iii) major-power media dominance. To mitigate these risks within a transparent and reproducible pipeline, we: (a) aggregate events at the country–year level to smooth short-term outlet shocks; (b) retain only records with unambiguous country identifiers (Actor1CountryCode/Actor2CountryCode) and standardized ISO codes; (c) use NumArticles (SNA) as the baseline proxy for media attention—capturing the breadth of coverage across distinct articles to avoid double-counting closely related signals; (d) apply within-year normalization when constructing NSII components to reduce sensitivity to year-to-year changes in global news volume; and (e) employ hotspot thresholds (CEN > 10,000; NSII > 200) strictly for cartographic labeling and descriptive counts. These steps do not eliminate all measurement bias, but they enhance comparability across countries and years and ensure that results are interpreted as comparative patterns of reported NTS activity rather than census-style totals. We also note that small variations of the labeling thresholds do not alter qualitative conclusions (see Sect. 3.3).

### Construction of nontraditional security impact index (NSII)

Based on the pre-processed data described in Sect. 3.1, this section details the construction of the Nontraditional Security Impact Index (NSII), a synthetic measure designed to capture the overall severity and significance of NTS issues faced by each country and the world.

Notation and Symbols. Let *M* denote the set of countries (indexed by *m*), and $T=\{2000,\dots ,2023\}$ the set of years (indexed by *t*). Let *G* be the set of NTS-relevant event types (indexed by *g*,$\left|G\right|=79$). When bilateral actors are referenced, *i* and *j* indicate the countries associated with Actor1 and Actor2 in GDELT. By using GDELT variables NumArticles, Goldstein Scale, and Average Tone as defined in Section “Literature Review”, a more accurate measurement of the event’s influence can be achieved. To ensure numerical stability we use two small constants:$\varepsilon ={10}^{-12}$ (to avoid division by zero in normalizations) and $\delta =1$ (to avoid $\mathrm{log}(0)$ in the global log transform). Based on this, a dynamic individual (single country) and overall (global) NSII is constructed by calculating the event impact and normalizing the event impact coefficient over years. The process involves three key steps:

1. Degree of Impact. It is measured by publication volume, reflects the attention received by a country’s NTS events, namely the event’s impact. The formula is as follows:
  1 $$A_{m} \left( {t,g} \right) = \mathop \sum \limits_{{i = m}} n_{{ij}} t,g + \mathop \sum \limits_{{j = m}} n_{{ij}} t,g - \mathop \sum \limits_{{i = j = m}} n_{{ij}} t,g$$
  where ${A}_{m}\left(t,g\right)$ represents the degree of NTS events influence of category $g$ involving country $m$ in year $t$, ${\mathrm{n}}_{{{\mathrm{ij}}}} {\mathrm{(t,g)}}$ indicates the search results, reflecting the number of news items on category $g$ NTS events between country $i$ and country $j$ in year $t$ (SNA), and $m$ denotes country’s serial number.
2. Normalizing the Event Impact Coefficient. The coefficient refers to the comprehensive assessment of Goldstein scales and average tone values of news events in the NTS domain occurring annually across countries. And it aims to measure the normalized impact coefficient of each country’s NTS events on itself and other countries by reducing irrelevant information interference. Alternative formulations (additive and PCA-based) were also tested and yielded consistent rank-order patterns, confirming that the multiplicative interaction primarily adjusts for media tone without distorting underlying event intensity. Firstly, normalize the Goldstein scales and average tone values separately, using the following formulas:
  2 $$\begin{array}{c}GS(g)=\left[x(g)-x{\left(g\right)}_{min}\right]/\left[x{\left(g\right)}_{max}-x{\left(g\right)}_{min}+\varepsilon \right]\end{array}$$
  3 $$\begin{array}{c}{T}_{m}\left(t,g\right)=\left[{y}_{m}\left(t,g\right)-y{\left(t,g\right)}_{min}\right]/\left[y{\left(t,g\right)}_{max}-y{\left(t,g\right)}_{min}+\varepsilon \right]\end{array}$$
  In the equation, $GS(g)$ represents the normalized “Goldenstein Scale” value of type $g$ NTS events that are unaffected by time. ${T}_{m}\left(t,g\right)$ denotes the normalized “Average Tone” value of type $g$ NTS events between country $i$ and country $j$ in year $t$. Both $x(g)$ and ${y}_{m}\left(t,g\right)$ are retrieval results, where $x(g)$ represents the “Goldenstein Scale” value of type g NTS events, $x{\left(g\right)}_{min}$ and $x{\left(g\right)}_{max}$ indicates the minimum and the maximum “Goldenstein Scale” value among NTS events, and these values are constants. A higher impact coefficient indicates greater severity of the event.
  Thus, the influence coefficient $I$ of type $g$ NTS events in year $t$ can be expressed as:
  4 $$I_{m}\left( {t,g} \right) = GS\left( g \right)*T_{m} \left( {t,g} \right)$$
  Furthermore, the normalized influence coefficient $\theta$ is as follows:
  5 $$\theta _{m}\left( {t,g} \right) = \frac{{I_{m}\left( {t,g} \right)}}{{\mathop \sum \nolimits_{{g = 1}}^{n} I_{m}\left( {t,g} \right) + \varepsilon }}$$
  where $~~\theta _{m}\left( {t,g} \right)$ represents the normalized influence coefficient of NTS events involving country $m$ in year $t$.
3. NSII. It is used to measure the comprehensive impact of NTS events for every country, as follows:
  6 $$NSII_{m} \left( t \right) = \mathop \sum \limits_{{g = 1}}^{n} \theta _{m} \left( {t,g} \right)*A_{m} \left( {t,g} \right)$$
  where $NSII_{m}(\mathrm{t})$ denotes the NTS impact index of country $m$ in year $t$, $n$ represents the number of types of NTS events selected in this paper, and n is equal to 79.

For ease of comparison and visualization, the logarithm of $NSII(t)$ is taken as the final index of NTS event impact. Therefore, the $NSII$ for the global in year $t$ can be expressed as follows:

7 $$\begin{array}{c}{NSII}_{global}\left(t\right)={\sum }_{m=1}^{a}{NSII}_{m}(\mathrm{t}),{\widetilde{NSII}}_{global}\left(t\right)=\mathrm{ln}({NSII}_{global}\left(t\right)+\delta )\end{array}$$

where ln denotes the natural logarithm (base *e*), *a* is the number of countries, *n* = 79 is the number of event types, and $\delta =1$ prevents $ln(0)$ when the annual global sum equals zero. A higher ${\widetilde{NSII}}_{global}\left(t\right)$ indicate greater harms of global NTS issues in year *t*.

Besides our multiplicative composite $I_{m} t,g$, we compute two theory-driven additive variants ${I}_{0.5}=0.5GS+0.5T$ and ${I}_{0.6}=0.6GS+0.4T$,and a data-driven PCA composite ${I}_{pca}={\lambda }_{GS}GS+{\lambda }_{AT}T$ using the first principal component of [GS, T] fitted on the whole sample. From Table A2, we can see the off-diagonal Pearson correlations among the four *I* variants are very high, and Spearman correlations are similarly high. For all variants, we keep our original pipeline: within (year × full event code) min–max normalization to $\theta$ (with $\theta =0.5$ when group variance is zero and denominator lower-bounded by $\varepsilon ={10}^{-12}$, exposure *A* set to SNA (article counts), and NSII aggregated as $NSII=\sum \theta *A$ at the country–year level. We report correlations across variants and threshold sensitivity (0.80/0.90/0.95) in the Supplementary Appendix (Table A2-A4).

### Analytical methods

To analyze the spatiotemporal evolution of global non-traditional security (NTS) issues, this study employed a combination of quantitative spatial analysis and visual analytics.

First, Geographic Information System (GIS) techniques were applied to visualize the global distribution patterns of NTS frequency (CEN) and impact (NSII). All maps were generated using ArcMap 10.7. Country-level NSII and CEN values were mapped annually to track changes in spatial distribution and identify areas with significant concentration. NSII and CEN values were divided into categories using natural breaks (Jenks method) based on their annual CEN and NSII values. Additionally, to evaluate the statistical significance of spatial clustering, the study conducted a Global Moran’s I spatial autocorrelation analysis annually from 2000 to 2023. This allowed for a formal assessment of whether the observed clustering patterns of CEN and NSII were statistically significant or random, which could help validate the temporal and spatial dynamics of global NTS hotspots and highlight the growing regional concentration of impact in recent years. In addition, phase boundaries are identified descriptively from aligned local extrema and slope-sign reversals in the global CEN and NSII series and are used for exposition rather than statistical inference. As a sensitivity check, shifting each breakpoint by ± 1 year leaves all qualitative findings (peak years, hotspot geography, and stage-wise summaries) unchanged.

Second, hotspot identification criteria were established based on empirical thresholds: countries with CEN > 10,000 were labeled as high-frequency zones, while those with NSII > 200 were identified as high-impact zones. These thresholds correspond to the upper quantile of observed values. As a sensitivity check, we also inspected adjacent cut points (CEN > 9,000 / 11,000; NSII > 180 / 220) and found no material changes in hotspot geography or stage-wise counts; we therefore retain the original thresholds for transparency and comparability.

Finally, a stage classification framework was developed to categorize global NTS evolution into five distinct periods (Initial, Outbreak, Transition, Easing, and New Normal), based on major inflection points in the global-level NSII and CEN curves.

## Results and analysis

### Temporal and spatial evolution of global NTS issues

**Fig. 1:**
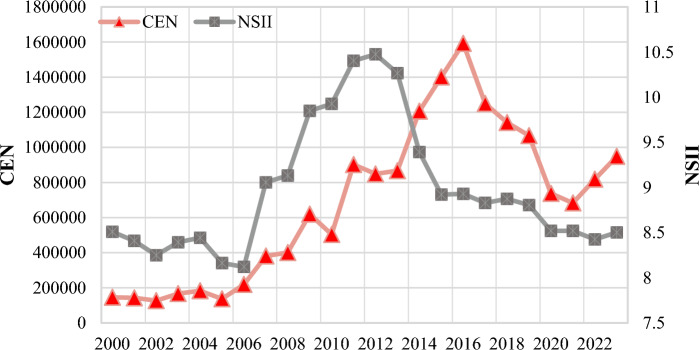
Time series graph of frequency and impact index of NTS event worldwide, 2000–2023. The left vertical axis represents the values of CEN (Event Frequency, measured in times), and the right vertical axis represents the values of NSII (Impact Index, ln-scale).

Since the beginning of the twenty-first century, international attention and concern towards NTS issues have gradually increased, driven by events such as the 9/11 attacks, climate change, pandemics, and the rise of cybersecurity threat^14,47,48^. Therefore, the year 2000 was chosen as the beginning year in this study to conduct statistical analysis on the frequency and impact indices of NTS events worldwide. Overall, the NSII showed a fluctuating upward trend prior to 2012, particularly experiencing a significant staircase-like rise from 2007 to 2012. It began to decline from 2013 onwards, sharply dropping by 15% (from 10.48 in 2013 to 8.92 in 2015). Subsequently, accompanied by a gradually slowing rate of decline, the NSII entered a relatively stable phase until a slight staircase decline in 2020, followed by a new stable period. During this period, NSII has approached the level in the early twenty-first century. While the curve shape of NTS frequency (denote as ‘CEN’ hereafter) closely resembles that of NSII, the peak of CEN occurred later in time (Fig. 1).

**Fig. 2:**
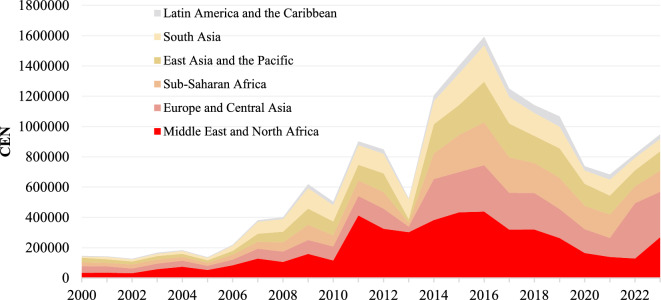
Total numbers of NTS events sort by region, 2000–2023. Regions are ordered from bottom to top based on their total event frequency (CEN) in 2016, from highest to lowest. A dark-to-light color gradient is used, with darker shades indicating regions with higher NTS event counts.

From a spatial perspective, as shown in Fig. A1, prior to 2006, NTS events with the high frequencies (CEN exceeding 10,000) or high impact indices (NSII greater than 200) were primarily distributed in individual countries. Between 2007 and 2012, the distribution of countries rapidly formed several cluster regions such as the Middle East, North Africa, and South Asia, with the Middle East and North Africa being the most severe (Fig. 2). And through Fig. A1 the distribution of countries with high NTS events frequencies and high impact indices evolved from isolated to multiple outbreaks. During the period, countries with high NTS events frequencies and those with high impact indices almost exhibited consistent distribution. By 2013, a differentiation in the distribution of countries began to emerge, with a sharp reduction in countries with high impact indices while countries with high frequency continued to increase, most notably in Sub-Saharan African countries. Since 2016, the number of countries with high impact indices have continued to decline, while those with high event frequencies have begun to decrease, and regional distributions have started to contract, particularly in the Middle East, North Africa, Sub-Saharan Africa, East Asia, and the Pacific region, with a notable decline in Sub-Saharan Africa south of the Sahara.

To objectively identify structural breaks in the time series of NSII and CEN indicators, we applied the Bai-Perron multiple breakpoint test (Bai & Perron, 2003) using the strucchange package in R. This method statistically detects the number and location of significant shift points in a time series without prior specification. The optimal number of breaks was determined based on the Bayesian Information Criterion (BIC). The statistically identified breakpoints were then cross-validated with major exogenous events (e.g., policy interventions, the COVID-19 pandemic) to ensure their substantive interpretability. This analysis revealed breakpoints in the years 2006 (95% CI: 2005–2007), 2014 (95% CI: 2013–2015), and 2020 (95% CI: 2019–2021), which guided our phase definition and largely corroborated the initial phase classification based on descriptive trends. Therefore, this paper divides the evolutionary process of global NTS issues into five stages: initial period (pre-2006), outbreak period (2007–2012), transitional period (2013–2016), easing period (2017–2021), and new normal (2022-present).

To statistically verify the existence of spatial clustering in NTS event distribution, we calculated the Global Moran’s I index for both the frequency (CEN) and impact index (NSII) of NTS events from 2000 to 2023 with a distance-based Euclidean weights matrix (Table A5). The results show that the spatial distribution of NTS impact became increasingly clustered after 2015, with NSII exhibiting significantly high Moran’s I values (e.g., 0.23 in 2015 and 0.20 in 2018; p < 0.001), indicating strong positive spatial autocorrelation. This suggests that countries with high NSII tend to be geographically proximate, forming persistent regional hotspots. In contrast, CEN presented relatively weaker clustering, and its Moran’s I values were generally lower and less stable, highlighting a more scattered pattern of event frequency. In addition, the Local Indicators of Spatial Association (LISA) analysis did not reveal extensive or statistically robust local clusters beyond the broad patterns already identified by the Global Moran’s I.

The intensifying spatial clustering of NSII underscores a shift from sporadic events to sustained regional crises with broader impacts. This further validates the rationale for distinguishing different evolutionary phases in the global NTS process and calls for geographically coordinated international responses.

### Characteristics of different stages during NTS issues evolutionary process worldwide

1. Initial period (pre-2006).

The initial phase is considered to be the period before 2006, during which the frequency and impact index of NTS events remained at a relatively low value (Fig. 1), indicating limited frequency and impact of NTS events worldwide. The quantitative analysis identifies the top five countries most impacted by NTS issues during this initial phase, ranked by their average NSII: Iraq, Palestine, Russia, Uganda and Nigeria. This period is considered the baseline before the widespread emergence of global NTS challenges.

As shown in Fig. A1, most countries had an impact index lower than 200, with only five countries exceeding this threshold during this period. Specifically, there were only two countries with an impact index greater than 200 from 2003 to 2006. On the contrast, the number of countries with CEN over 10,000 increased from two to six during the same period. Overall, this stage was characterized by sporadic outbreaks, with only a few countries facing relatively serious NTS events, but without establishing a widespread pattern.

In this period, Iraq stood out significantly in terms of the frequency and impact index of NTS events. Especially in 2003 there were fluctuations in NSII and CEN worldwide, largely attributed to the outbreak of Iraq War. The period from 2003 to 2006 was marked by complexity and instability due to the Iraq War, which not only triggered NTS concerns such as humanitarian crises, religious conflicts, and security issues, but also provided fertile ground for terroris^49^. Frequent occurrences of terrorist attacks, suicide bombings, and other forms of terrorism proliferated in Iraq and surrounding areas, leading to the rise of new terrorist organization^50^. The Iraq War was not merely a conventional military conflict but a comprehensive challenge that severely impacted the production, livelihoods of the Iraqi people, and stability in the Middle East. Consequently, Iraq exhibited a relatively high impact index in NTS compared to other surrounding countries during this period. Overall, the global in this stage showed relatively low impact indices in NTS, attributed not only to fewer total NTS events but also to the underdeveloped state of media technology at the time, which limited the fidelity, speed, and scope of event reporting and dissemination.

(2) Outbreak period (2007–2012).

From 2007 to 2012 marked an outbreak phase in the evolution of NTS issues worldwide. CEN and NSII rose sharply, marking a global surge in the frequency and severity of NTS incidents. This phase was dominated by specific regional crises. The top five countries by average NSII were Iraq, Afghanistan, Pakistan, Syrian Arab Republic and Egypt. The concentration of these hotspots in South Asia and the Middle East underscores the region-specific nature of the outbreak.

During this period, both the number and impact index of NTS events surged synchronously, with the impact index growing more intensively than the frequency of events, reaching its peak in 2012. In 2007, the number of countries with NSII exceeding 200 exceeded 5, and this figure increased by 28 between 2007 and 2012. The countries experiencing event frequencies surpassing 10,000 surged from 8 in 2007 to 21 in 2012. Throughout this stage, the distribution of countries exhibiting high frequencies and impact indices transitioned from isolated incidents to multiple outbreaks, giving rise to several clustered regions including the Middle East, North Africa, and South Asia.

**Fig. 3:**
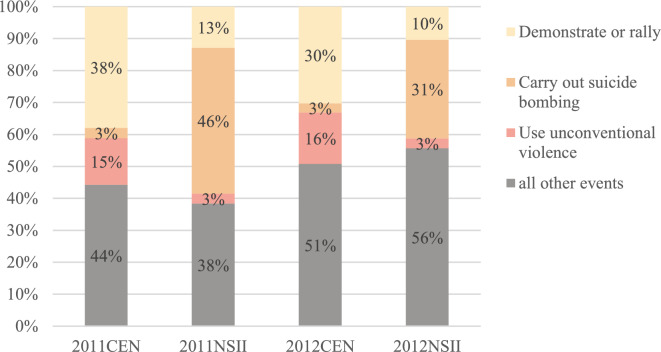
Total numbers and influence index of NTS event,2011–2012.

In this period, the global financial crisis, frequent conflicts and terrorism in regions such as Afghanistan, Syria, and Libya, accompanied by recurring natural disasters, along with energy security and transnational crime, posed significant threats to the security and stability of the global. Hence, both the number and impact index of NTS events surged synchronously; the mean annual NSII during this phase was 9.81, a 17.78% increase from the previous phase’s mean of 8.33, while the mean annual CEN reached 609,649, a 280% increase (Fig. 1). Events such as demonstrating or rallying (code 141), carrying out suicide bombing (code 1831), and using unconventional violence (code 180) accounted for approximately half of the overall occurrences and impact index globally (Fig. 3). Demonstrating or rallying accounted for over 30% of all events, ranking second in NSII among all type of events. These events were primarily concentrated in the Middle East and North Africa, largely influenced by the spread of the ‘Jasmine Revolution’ in the Arab region^51^. The highest NSII was observed in suicide bombings (i.e.one kind of terrorism activities). In 2011, their cumulative impact index nearly equaled half of the total. Afghanistan and Pakistan were hotspots for such events, collectively accounting for more than half of all occurrences (Table 3). Additionally, the increasing NSII during this period was also influenced by the rapid development of news media, which amplified the impact of events.

**Table 3:** Percentage distribution of three major kinds of NTS events,2011&2012.

Demonstrate or rally		Use unconventional violence		Carry out suicide bombing	
Country	Percentage	Country	Percentage	Country	Percentage
SYR	10.8	SYR	11.4	AFG	43.6
EGY	9.7	AFG	6.9	PAK	15.6
CHN	5.7	LBY	6.0	IRQ	9.8
PAK	4.8	PAK	5.3	NGA	4.9
YEM	4.1	EGY	4.7	SYR	4.4
RUS	4.0	CHN	4.0	YEM	3.9
LBY	3.8	PSE	3.9	RUS	3.7
IRN	3.5	RUS	2.8	SOM	3.1
TUN	3.4	NGA	2.6	IDN	1.4
PSE	3.3	IRQ	2.3	EGY	1.3
All other countries	46.8	All other countries	50.2	All other countries	8.1
Total	100.0	Total	100.0	Total	100.0

(3) Transitional period (2013–2016).

This stage is characterized by a continued increase in the number of NTS events, while the impact index of these events has begun to decline. A shift in spatial patterns is evident in the top-ranked countries for this transitional phase: Iraq, Egypt, Pakistan, Nigeria and Afghanistan. The emergence of Syria and Nigeria in the top five, alongside the persistent presence of Afghanistan and Iraq, signals the geographic expansion and intensification of conflicts in the Middle East and the rise of significant threats in Sub-Saharan Africa.

The number of countries with impact indices exceeding 200 sharply decreased from 32 to 1 during this stage. However, the number of countries with more than 10,000 events continued to increase, rising to 37 from the previous period, with Sub-Saharan African countries experiencing the most rapid growth. The Middle East, North Africa, and South Asia remained hotspots for NTS events, showing a persistent upward trend. This trend expanded towards the east, south, and north from these centers. As depicted in Fig. 2, Sub-Saharan Africa, East Asia and the Pacific, as well as Europe and Central Asia, witnessed rapid increases in the frequency of NTS events, reaching a peak in 2016.

**Fig. 4:**
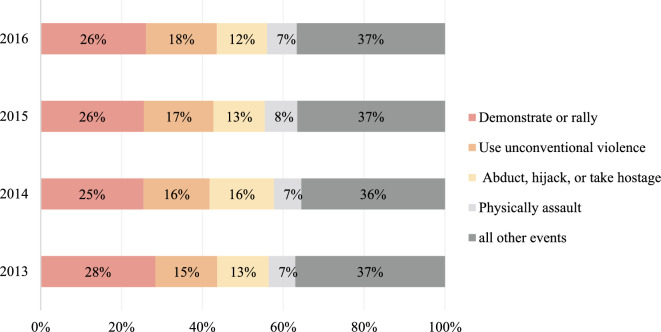
Total numbers of non-traditional security events sort by event code, 2013–2016.

During the transitional phase, challenges such as the lingering effects of the European sovereign debt crisis, the rise of major emerging economies, and declining commodity prices introduced uncertainty to the global economic situation. The Syrian civil war and instability in Iraq heightened tensions in the Middle East. In such circumstances, the proliferation of terrorism, epitomized by groups like the Islamic State (IS), expanded their influence opportunistically in the Middle East and beyond, resulting in large-scale acts of violence and terrorist attacks. According to the Global Terrorism Index Report by the Institute for Economics & Peace (IEP), the number of deaths due to terrorism in 2016 reached the highest level in nearly a decade, with Iraq, Nigeria, and Afghanistan alone accounting for more than half of global fatalities. Accordingly, the NTS events became increasingly frequent in multiple countries, experiencing a rapid surge in 2015 and peaking in 2016. The four most common types of events during this period were “Demonstrate or rally”, “Use unconventional violence”, “Abduct, hijack, or take hostage” and “Physically assault” (Fig. 4).

(4) Easing period (2017–2021).

From 2017 to 2021, there was a phase of alleviation in the evolution of NTS issues globally. The easing phase is characterized by a notable decline in the intensity of NTS impacts. The top five countries, while still experiencing significant issues, had lower average NSII values compared to previous phases: Palestine, Afghanistan, Pakistan, Zimbabwe and China. The gradual reduction in NSII values across these top countries reflects the beginning of a global alleviation trend.

The number of NTS events markedly declined, and the NSII stabilized. There were almost no countries with an impact index exceeding 200 during this phase, with 99% of sample countries having an impact index below 100 by 2021, indicating that the influence of events had reverted to regional or national scopes. As shown in Table 4, the number of countries experiencing more than 10,000 events decreased from 31 to 15. Regions witnessing reduced intensity included the Middle East and North Africa, Sub-Saharan Africa, East Asia, and the Pacific, particularly Sub-Saharan Africa.

**Table 4:** The regional distribution of NTS events exceeding 10,000 occurrences, 2017–2021.

	2017	2018	2019	2020	2021
South Asia	2	2	2	2	2
East Asia and the Pacific	5	4	5	2	2
Sub-Saharan Africa	14	12	14	7	5
Middle East and North Africa	5	5	5	4	2
Europe and Central Asia	5	4	3	4	4
Latin America and the Caribbean	0	0	1	0	0
Total	31	27	30	19	15

(5) New normal (2022-present).

Since 2022, the global NTS landscape appears to have entered a tentative “New Normal”, both indices exhibited relative stabilization, though variations persisted across regions. The New Normal is defined by a continued decline in the severity of NTS events. The top five countries by average NSII in this most recent period are Ukraine (primarily due to the traditional security conflict and its NTS repercussions), Palestine, Russia, Pakistan and Afghanistan. The entry of Ukraine into the list is a key development, illustrating how traditional security conflicts can drive and transform NTS challenges.

The quantitative analysis confirms the emergence of a New Normal phase post-2021, characterized by stabilized, low-level NTS activity. A Mann–Kendall trend test confirms no significant trend since 2020 (p > 0.05), reinforcing stability. Spatially, high-impact events remain constrained, with fewer than three countries per year exceeding NSII 100, starkly contrasting the over 30 countries during the Outbreak period. A 2023 fluctuation in CEN (> 10,000) is attributed to the Russia-Ukraine war’s ripple effects but did not alter the stable NSII, underscoring the phase’s resilience.

In the long term, NTS issues are likely to coexist with humanity and may not completely disappear. These issues are becoming increasingly complex, transcending national borders and defying simple military solutions. Over time, with economic and global developments, there will be new NTS challenges. Globalization intensifies some of these issues but also presents opportunities for their resolution. It is crucial means for addressing NTS issues to respect global trends and enhance international cooperation.

### Causes of stage evolution of NTS issues globally

#### Initial period (pre-2006)

Following the end of the Cold War, adjustments in the global geopolitical landscape and economic map highlighted the rise of non-military threats, which posed challenges to the national and social stability. Many countries no longer viewing other countries as threats. Consequently, the national security strategies of major powers shifted to encompass dimensions beyond mere military security. Following the September 11 attacks, terrorism became the primary NTS issue. In response, the United States launched a global war on terror, initiating the Afghanistan War in 2001 and subsequently invading Iraq in 2003 under the pretext of weapons of mass destruction. This global counterterrorism strategy drew significant international attention to terrorism. For the global, this period represented the initial stage of NTS issues evolution and the nascent phase of understanding these issues. At that time, globalization was still in its early stages, and NTS issues had not yet become complex in their types or manifestations, nor had they exhibited cross-domain or comprehensive characteristics. Consequently, the understanding of NTS issues among countries was relatively limited, marking an early phase in their evolutionary development.

#### Outbreak period (2007–2012)

Many countries involved in the sample are developing nations that, at that time, were undergoing accelerated urbanization and industrialization. This shift, characterized by the migration of rural labor to cities, led to significant changes in social structure and exacerbated social conflicts and instability factors^52^. As economic development progressed rapidly, various social issues begun to emerge, including unstable political system, aging populations, inadequate education and healthcare, environmental pollution, and scarce resources^53–55^. These issues impacted local residents’ livelihoods and development to varying degrees, posing potential threats to social stability.

Geopolitical tensions and regional conflicts further increased uncertainties and risks, such as turmoil in the Middle East, disputes between China and Philippines in East Asia and the Pacific, and conflicts between India and Pakistan in South Asia^56–58^. Terrorism has exploited these conditions, threatening regional security. Additionally, the accelerated pace of globalization has made international relations increasingly complex, presenting multiple challenges. Notably, the global economic turmoil triggered by the 2008 financial crisis is associated with economic instability and trade contraction worldwide, plummeting oil prices, reduced foreign direct investment, increased unemployment rates, heightened fiscal pressures on governments, and shocks to economic stability and social order^59,60^. Moreover, the rapid development of news media technology has facilitated faster information dissemination, expanding the depth and breadth of news coverage. The rise of online media platforms, such as X (formerly known as Twitter), has enriched information sources and accelerated the evolution of NTS issues.

#### Transition period (2013–2016)

NTS issues such as terrorism, religious extremism, transnational crime, piracy, natural disasters, energy and economic crises, and climate change continue to pose significant threats to human security during the transition period. Inter-state conflicts not only fail to alleviate these threats but often exacerbate them, necessitating diverse diplomatic approaches. Almost countries have recognized these challenges and initiated multi-sectoral cooperation on NTS issues, including climate cooperation^61^, counter-terrorism cooperation^62^, transnational crime cooperation^63^, bilateral defense cooperation agreements^64^, and limited cybersecurity cooperation^65^.

Zografos, et al.^66^ identify structural violence, often linked to economic and national development issues, as a major source of human insecurity. The Belt and Road Initiative (BRI) proposed by Chinese president Xi Jinping in 2013, has introduced new opportunities and impetus for economic development and cooperation through outward investments in these regions^67^. However, as of June 30, 2016, only 56 countries had joined the initiative, fewer than half the sample size of this study. The period from 2013 to 2016 primarily served as a preparatory phase for the BRI, requiring significant time to establish, implement and refine mechanisms for NTS cooperation, with results yet to be fully realized.

Simultaneously, changes in major power relations, regional hotspots, and the international trade system have transformed the international political landscap^68–70^. It is trending towards multipolarity and increasing complexity, which significantly influences the transitional phase of the evolution of NTS issues. Furthermore, advancements in media technology, enhanced national risk management capabilities, and increased public awareness contribute to reducing panic and mitigating the negative impacts of events.

#### Easing period (2017–2021)

Driven by emerging technologies such as artificial intelligence, blockchain, and cloud computing^71^, China continued to sustain rapid economic growth, despite fluctuations due to the COVID-19 pandemic, remaining a primary engine of global economic growth. Climate change and sustainable development became focal points of global concern, prompting increased investment and cooperation among nations in clean energy and environmental technologies, thereby facilitating the global economic transition towards sustainability. Organizations such as the United Nations, the European Union, and the Arab League have actively mediated and facilitated dialogue and reconciliation on issues like the Israeli-Palestinian conflict and the Syrian civil war, promoting intra-regional dialogue and peace. What’s more, China has facilitated the restoration of diplomatic relations between Saudi Arabia and Iran, and has jointly participated with Middle Eastern countries in NTS governance, safeguarding the interests of developing countries.

With the advancement of the BRI, as of November 2021, China has signed cooperation agreements with 140 countries, ( XinHuaNet. To build a path of opportunity leading to common prosperity共建通向共同繁荣的机遇之路——习近平总书记谋划推动共建 “一带一路”述评[EB/OL].www.gov.cn/xinwen/2021-11/19/content_5651805.htm) closely approaching the sample size of this study. As BRI cooperation expanded, we observe contemporaneous declines in CEN/NSII in many participating countries. These patterns are associations rather than causal effects and may also reflect concurrent factors. Within the framework of the BRI, a series of international cooperation mechanisms and platforms, such as the Belt and Road International Cooperation Summit Forum, have been established providing a platform for dialogue, exchange, and cooperation in the security field, contributing to collective efforts to address non-traditional security threats. Through increased dialogue and consultation, political mutual trust among BRI countries has been enhanced, helping to mitigate potential security risks and conflicts. When facing transnational security threats such as the COVID-19 pandemic, terrorism, transnational crime, and climate change, the BRI countries are more inclined to respond through cooperation rather than unilateral actions. Additionally, the dual-edged role of social media^72^ is widely recognized, with both the public’s ability to discern news and the government’s ability to control public opinion strengthening, thereby limiting the escalation and spread of events to some extent.

#### New normal period (2022-present)

Since the implementation of the BRI, it has spurred global economic recovery and growth, providing new impetus for development. The convening of the Third Belt and Road Forum for International Cooperation in 2023 marked a new milestone, fostering closer ties among BRI countries and establishing mechanisms for shared development. Economic issues are often the root cause of many NTS challenges. Economic factors are often intertwined with many non-traditional security (NTS) challenges, as improved development conditions can mitigate poverty and social instability, thereby potentially lowering the risks of terrorism, extremism, and other social crises. Within this context, closer cooperation under the BRI framework may have contributed—alongside other global processes such as post-pandemic recovery and shifts in international engagement—to enhanced economic activity and stability in several regions. However, such relationships should be understood as associations rather than direct causal effects.

According to the multilateral cooperation outcomes documented at the Third Belt and Road Forum for International Cooperation, ( http://www.beltandroadforum.org/n101/2023/1018/c134-1211.html) future efforts will further strengthen government cooperation in areas such as green finance, natural disasters, energy security, food security, maritime ports, cultural tourism, and other fields related to NTS challenges. These cooperative mechanisms may correlate with the observed stabilization of NTS indicators in recent years, but this interpretation remains provisional and warrants further empirical verification through comparative and longitudinal analyses.

The broader evolutionary trajectory of global NTS issues also reveals emerging characteristics such as intensified confrontation, increased transnational diffusion, and blurred boundaries between traditional and non-traditional security domains. Many NTS challenges originate from or interact with conventional security concerns, underscoring the need for integrated and cooperative governance frameworks. International cooperation—including, but not limited to, initiatives such as the BRI—may thus represent one potential pathway toward the collective management of NTS risks. Nevertheless, this study refrains from inferring causation and views these observed overlaps as indicative of broader global cooperation dynamics that merit continued examination in future research.

Notwithstanding its contributions, this study has several limitations that warrant emphasis. Substantively, the evidence is observational and development-focused: results reflect global patterns within a sample dominated by BRI participants and other low- and middle-income economies and should not be interpreted as causal. Methodologically, our metrics (CEN and NSII) are media-derived, which introduces potential endogeneity between actual events and their mediated visibility; coverage can be amplified in geopolitically salient regions or where Western interests are involved, while under-reported locales may see their true scale understated. Although deduplication and conservative actor-coding rules are applied, reporting/coverage biases may persist across countries and time, and GDELT’s event-classification limits mean some common NTS phenomena are not captured; moreover, heterogeneity in media ecosystems (language coverage, access, editorial practices) can affect cross-country comparability despite normalization steps. In addition, our hotspot thresholds and phase boundaries are descriptive heuristics (with sensitivity checks) rather than formal identification devices, and spatial summaries depend on the chosen distance-based weights. Taken together, these caveats counsel cautious interpretation and frame our findings as the global media footprint of NTS activity—a consequential dimension in its own right—while pointing to future work that triangulates media-based indicators with alternative sources (e.g., satellite imagery, NGO reports, official statistics) to better separate event signals from media amplification.

## Conclusion

Using nontraditional security impact index (NSII) and the NTS events frequency , this study examined the global evolution of non-traditional security (NTS) issues from 2000 to 2023 based on the GDELT database. Spatiotemporal characteristics were analyzed through GIS-based spatial analysis, revealing how the frequency and intensity of NTS events evolved and clustered across regions over time.

Across two decades, NTS dynamics followed a broad surge–decline–stabilization trajectory, which can be summarized into five approximate phases from initial emergence to a tentative stabilization after 2022. While descriptive curve segmentation and structural break tests both suggest an easing trend since 2017, the evidence for a “New Normal” remains preliminary, given the limited post-2022 data window. Longer time series will be needed to verify whether this pattern represents a sustained stabilization or a short-term plateau.

The evolution of NTS activity reveals increasing regional concentration, particularly in the Middle East, Sub-Saharan Africa, and parts of South and East Asia, followed by partial contraction in the late 2010s. The overall decline in intensity after 2017 coincides with multiple global developments—including expanded regional cooperation under initiatives such as the Belt and Road Initiative (BRI), post-crisis economic adjustments, and shifts in international engagement patterns. These trends should be interpreted as coinciding processes rather than causal effects, and the observed associations remain hypotheses for future testing.

The findings highlight the importance of multilateral cooperation frameworks for managing complex transnational risks. Economic development, disaster preparedness, environmental resilience, and information transparency may jointly contribute to mitigating NTS vulnerability, underscoring the need for cross-sectoral governance and data-driven monitoring systems. Rather than prescriptive advocacy, these implications point to potential pathways for improving early-warning capacity and global coordination.

Several limitations must be acknowledged. First, the study relies on media-derived data, which reflect patterns of news salience rather than objective event magnitude. Western and English-language media remain dominant in GDELT, leading to potential underrepresentation of low-coverage regions. Second, event severity is inferred from textual sentiment and Goldstein scores without direct calibration to physical or human losses. Third, coverage unevenness means that missing or sparse years in some countries may reflect reporting gaps rather than genuine absence of NTS activity.

Future research should aim to triangulate media-based measures with alternative datasets, such as ACLED (for conflict events), EM-DAT (for disasters), and WHO outbreak reports, to validate and refine the NSII framework. Methodological extensions could include spatial regression models (e.g., SAR, SEM) to examine diffusion and contagion dynamics, as well as causal identification strategies such as difference-in-differences or event-study designs around major global shocks. Such work would enhance the robustness and explanatory power of global NTS analysis.

## Supplementary Information

Supplementary Information.

## Data Availability

The data that support the findings of this study are available from the corresponding author, Shiyue Li, upon reasonable request.
