# Supplementary material for: Spatiotemporal analysis of nontraditional security issues evolution globally: evidence from news big data
Source: Sci Rep. 2026 Mar 11;16:13126. doi: 10.1038/s41598-026-42600-1 (PMC13100194; doi:10.1038/s41598-026-42600-1)
Supplement: Supplementary file 1 — Supplementary Information. [file 41598_2026_42600_MOESM1_ESM.docx]

Appendix


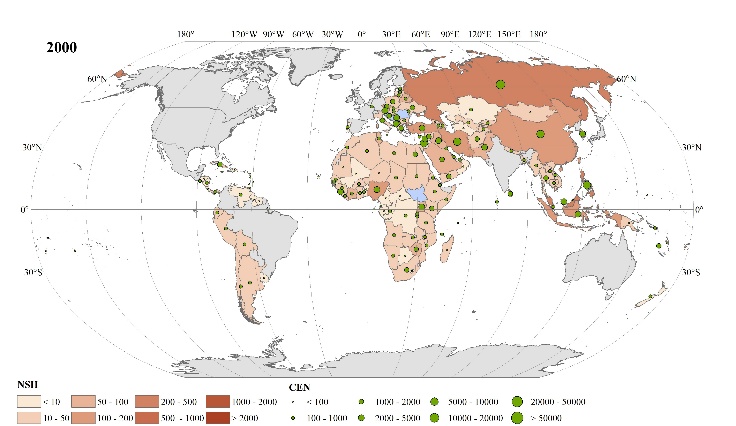

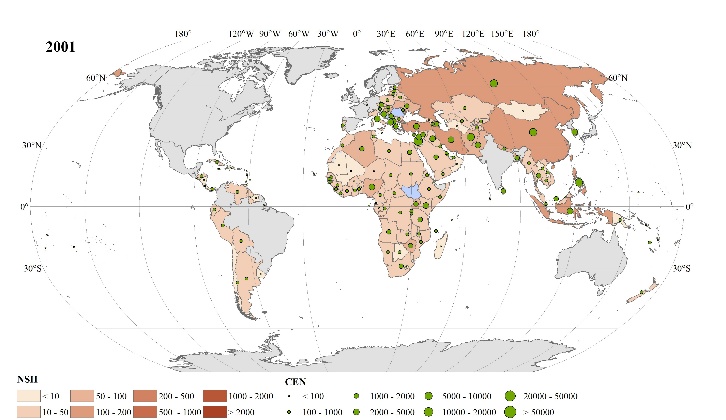


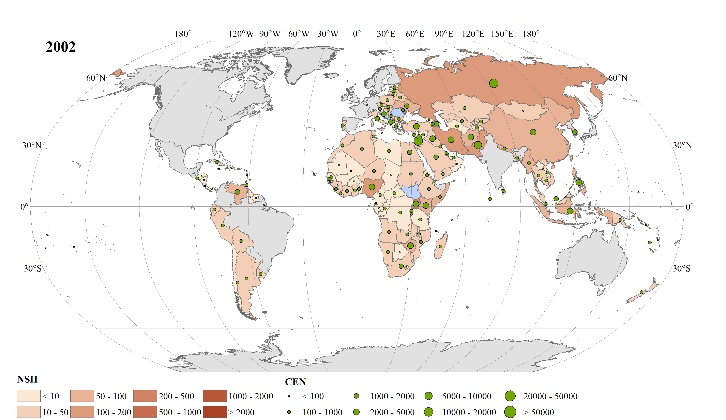

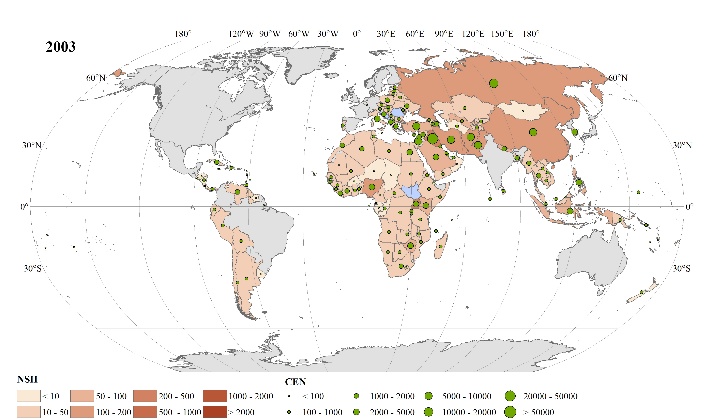


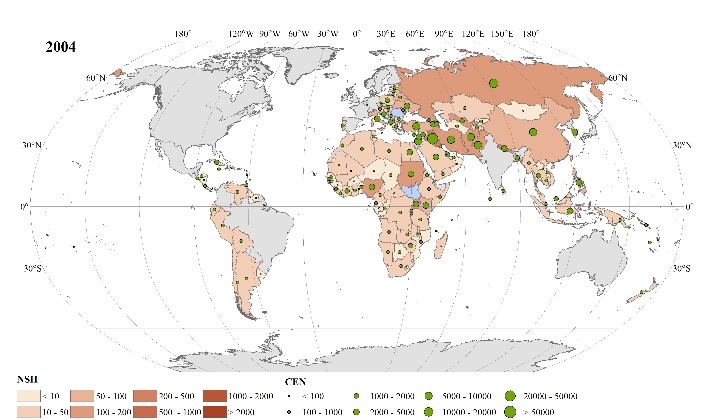

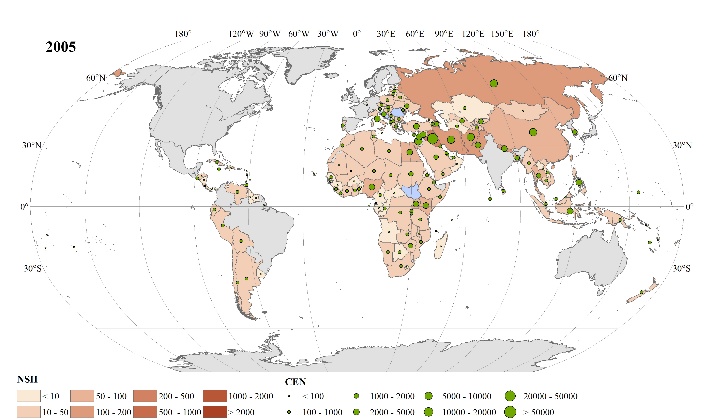


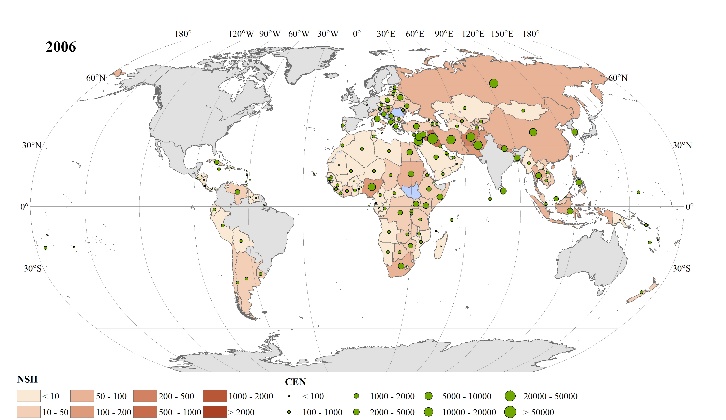

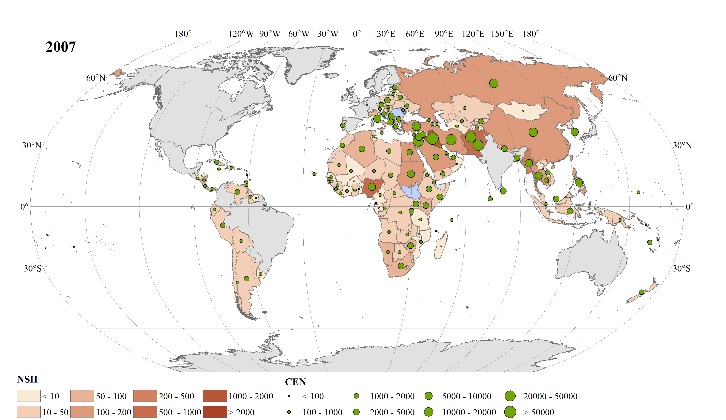


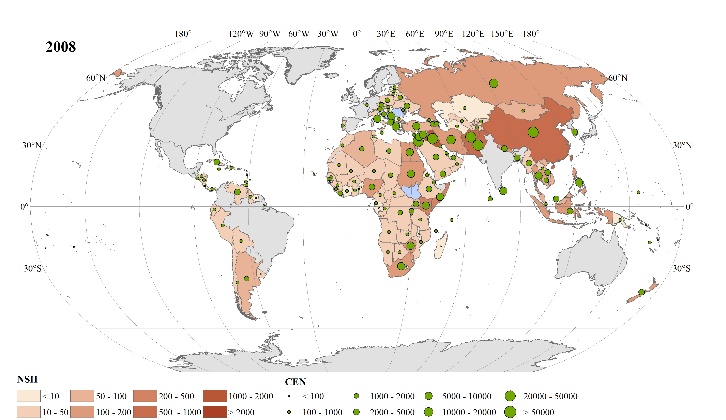

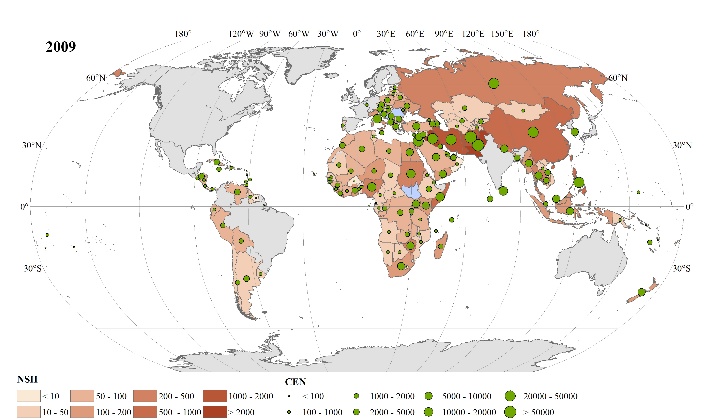


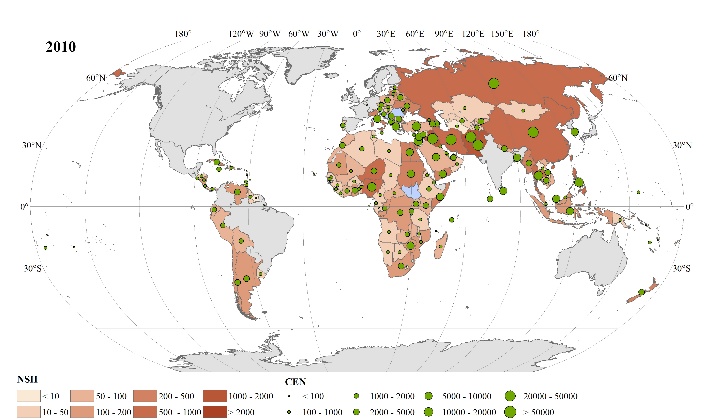

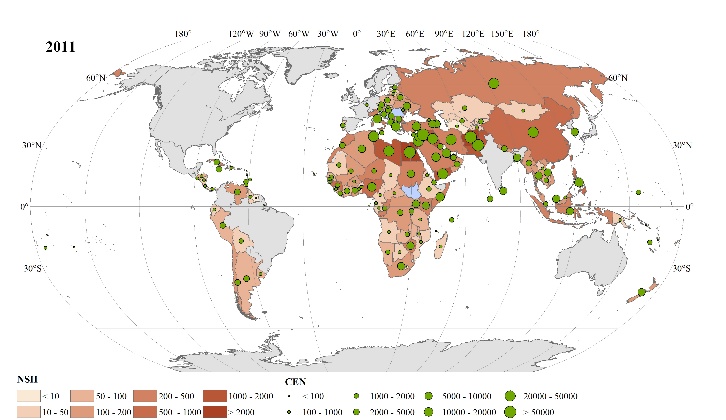


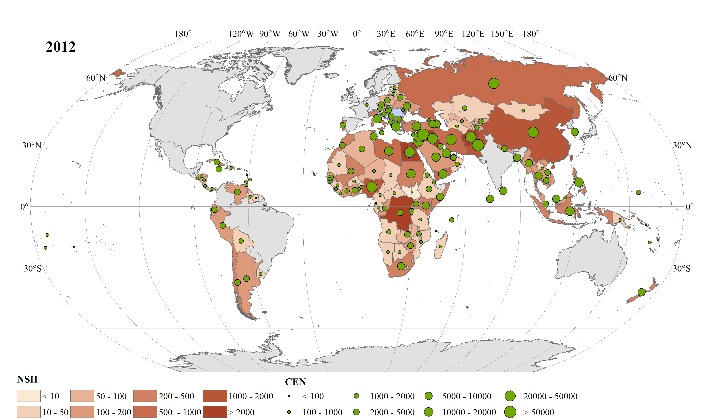

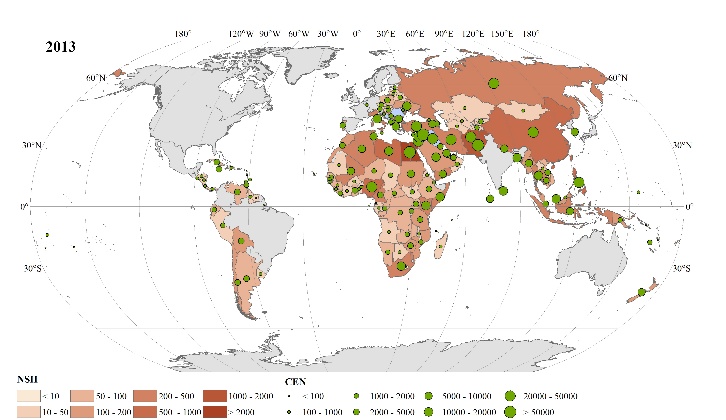


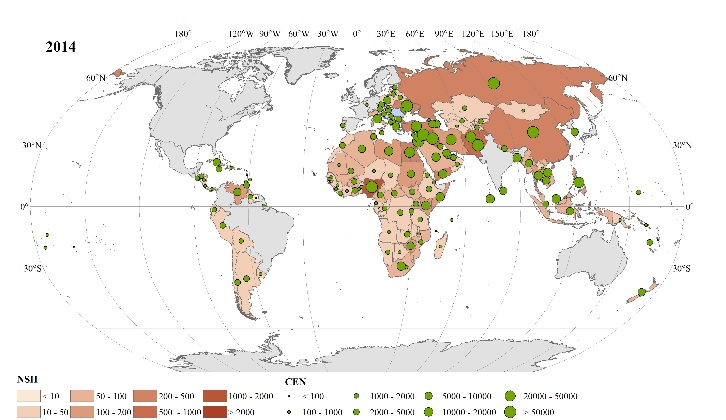

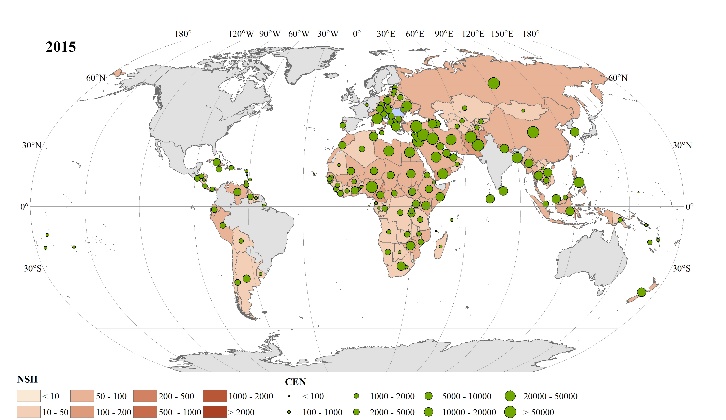


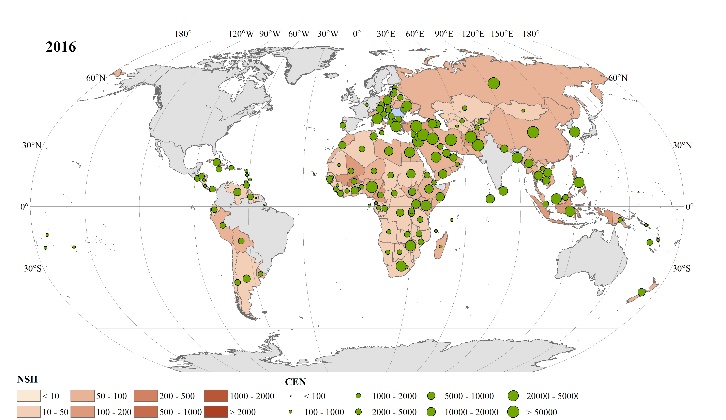

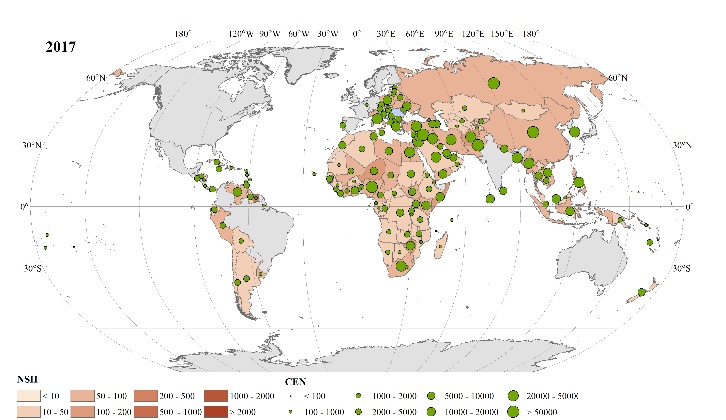


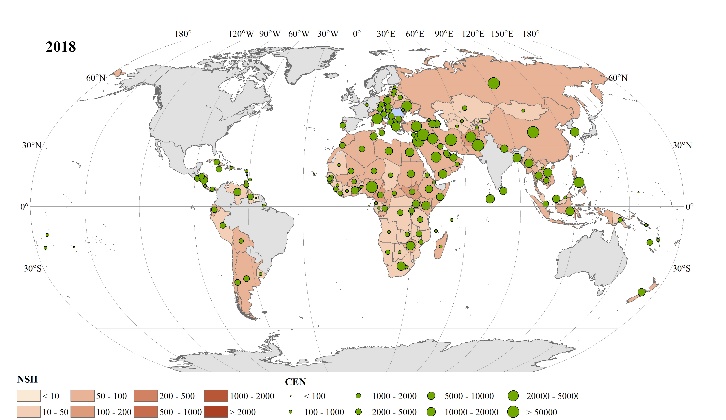

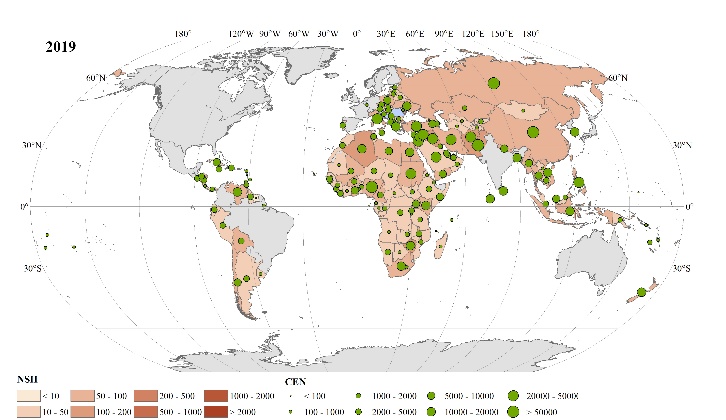


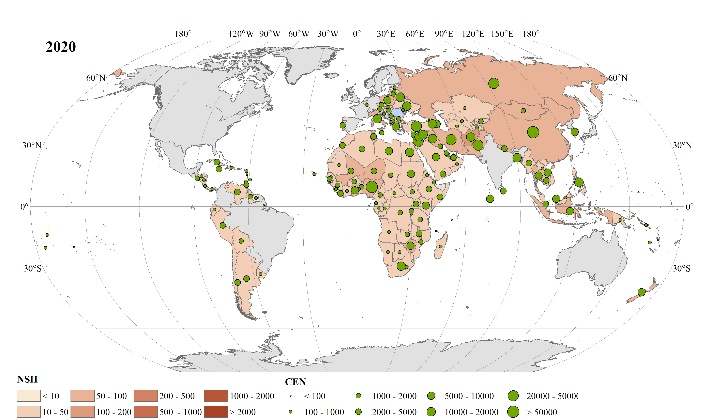

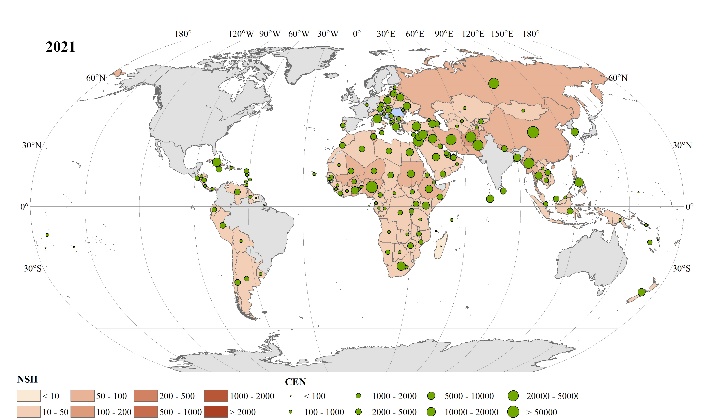


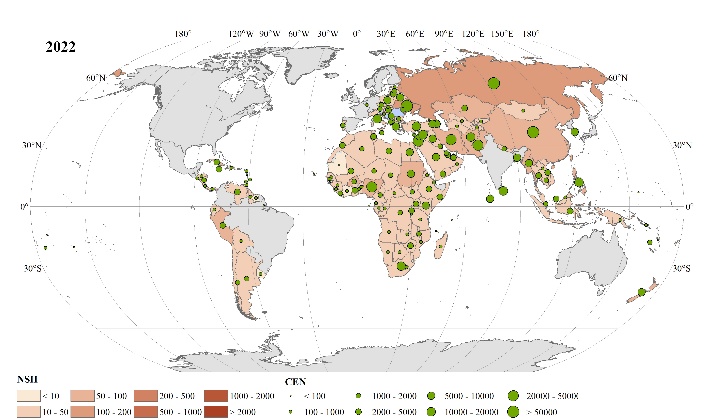

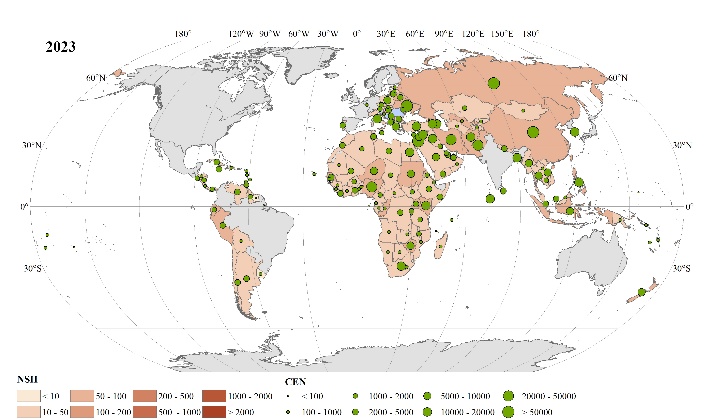


Fig.A1 Spatial distribution of the Non-Traditional Security Impact Index (NSII) and Event Frequency (CEN), 2000-2023. Maps were generated in ArcMap 10.7 (ESRI Inc., Redlands, CA). NSII and CEN values were calculated from GDELT, as described in Methods. Data are classified using the Jenks natural breaks method into eight quantiles. A sequential color gradient from light red (low values) to dark red (high values) is used, employing a color-vision-deficiency-friendly palette. The map is presented in the WGS 1984 geographic coordinate system. The world administrative boundary layer was sourced from Natural Earth (naturalearthdata.com). Hotspots defined as CEN > 10,000; NSII > 200.

Table A1 The Selected NTS Event Types from CAMEO Event and Actor Codebook

| CAMEO | Name | Description |
| --- | --- | --- |
| 103 | Demand material aid, not specified below | Require, demand provision of material assistance not otherwise specified. |
| 1031 | Demand economic aid | Require, demand provision of economic assistance. |
| 1033 | Demand humanitarian aid | Require, demand provision of humanitarian aid. |
| 104 | Demand political reform, not specified below | Require, demand political change not otherwise specified. |
| 1041 | Demand leadership change | Require, demand change in leadership or power |
| 1042 | Demand policy change | Require, demand change in any particular policy |
| 1043 | Demand rights | Require, demand provision or expansion of social, political, or other rights. |
| 1044 | Demand change in institutions, regime | Require, demand major institutional, constitutional, or regime change |
| 1051 | Demand easing of administrative sanctions | Require, demand that target relaxes administrative restrictions. |
| 1052 | Demand easing of political dissent | Require, demand that target stops political protest activities. |
| 1054 | Demand easing of economic sanctions, boycott, or embargo | Require, demand that target lifts or eases economic sanctions, boycott, or embargo. |
| 1055 | Demand to allow international involvement (non-mediation) | Require, demand that target allow access to international actors, such as observers, humanitarian agencies, and peacekeeping forces. |
| 1122 | Accuse of human rights abuses | Allege, charge the target with, or blame for human rights violations, such as arbitrary detentions for prosecutions, torture, and slavery. |
| 1125 | Accuse of espionage, treason | Allege, charge the target with, or blame for spying, espionage, or treason. |
| 1211 | Reject economic cooperation | Refuse to engage in or expand economic ties |
| 1213 | Reject judicial cooperation | Refuse to engage in or expand cooperation in judicial matters. |
| 1214 | Reject intelligence cooperation | Refuse to engage in or expand cooperation in intelligence or information sharing. |
| 1221 | Reject request for economic aid | Refuse to extend financial assistance |
| 1223 | Reject request for humanitarian aid | Refuse to extend humanitarian assistance. |
| 123 | Reject request or demand for political reform, not specified below | Refuse to institute political change not otherwise specified. |
| 1231 | Reject request to change leadership | Refuse to change leadership or relinquish power |
| 1232 | Reject request to change policy | Refuse to change a given policy |
| 1233 | Reject request for rights | Refuse to provide or respect social, political, economic or other rights and freedoms. |
| 1234 | Reject request for change in institutions, regime | Refuse to make fundamental political changes, such as moving from one type of a political system to another and reforming political institutions or key laws. |
| 124 | Refuse to yield, not specified below | Reject requests, refuse, or decline to yield not otherwise specified. |
| 1241 | Refuse to ease administrative sanctions | Reject requests, refuse or decline to ease administrative sanctions, such as censorship, curfew, state of emergency, and martial law |
| 1242 | Refuse to ease popular dissent | Reject requests, refuse, or decline to reduce or stop political protest activities, such as demonstrations and rallies. |
| 1243 | Refuse to release persons or property | Reject requests, refuse, or decline to release or return persons or property. |
| 1244 | Refuse to ease economic sanctions, boycott, or embargo | Reject requests, refuse, or decline to reduce or eliminate economic sanctions, boycotts, or embargoes. |
| 126 | Reject mediation | Refuse involvement of mediators or mediation initiatives. |
| 127 | Reject plan, agreement to settle dispute | Reject a proposal or request for a final, comprehensive settlement, peace  proposal, or resolution. |
| 1311 | Threaten to reduce or stop aid | Threaten to reduce or stop providing material aid. |
| 1312 | Threaten to boycott, embargo, or sanction | Threaten to restrict normal economic interactions by imposing sanctions, boycotts, or embargoes |
| 1313 | Threaten to reduce or break relations | Threaten to reduce or formally sever ties. |
| 133 | Threaten political dissent | Threaten to mobilize or engage in actions of political dissent such as protest demonstrations, hunger strikes, strikes or boycotts, physical obstructions into buildings or areas, and riots. |
| 1383 | Threaten unconventional attack | Threaten to use unconventional violence, including terrorist activities. |
| 1385 | Threaten unconventional mass violence | Threaten to use force potentially affecting large masses of people, including the use of weapons of mass destruction (nuclear or chemical-biological radiological attacks), mass expulsions or killings, as well as ethnic cleansing |
| 141 | Demonstrate or rally, not specified below | Dissent collectively, publicly show negative feelings or opinions; rally, gather to protest a policy, action, or actor(s). |
| 1411 | Demonstrate or rally for leadership change | Dissent collectively, gather, or rally demanding leadership change. |
| 1412 | Demonstrate or rally for policy change | Dissent collectively, gather, or rally demanding policy change. |
| 1413 | Demonstrate for rights | Dissent collectively, gather, or rally demanding political, social, economic, or other rights. |
| 1414 | Demonstrate for change in institutions, regime | Dissent collectively, gather, or rally demanding major institutional, constitutional, or regime change. |
| 142 | Conduct hunger strike, not specified below | Protest by refusing to eat until certain demands are met, not further specified. |
| 1421 | Conduct hunger strike for leadership change | Refuse to eat until demands for leadership change are met |
| 1422 | Conduct hunger strike for policy change | Refuse to eat until demands for policy reform are met. |
| 1423 | Conduct hunger strike for rights | Refuse to eat until demands for political, social, economic, or other rights are met. |
| 1424 | Conduct hunger strike for change in institutions, regime | Refuse to eat until demands for major institutional, constitutional, or regime change |
| 143 | Conduct strike or boycott, not specified below | Protest by refusing to work or cooperate until certain demands are met, not specified further |
| 1431 | Conduct strike or boycott for leadership change | Refuse to work or cooperate until demands for leadership change are met |
| 1432 | Conduct strike or boycott for policy change | Refuse to work or cooperate until demands for policy reform are met. |
| 1433 | Conduct strike or boycott for rights | Refuse to work or cooperate until demands for political, social, economic, or other rights are met. |
| 144 | Obstruct passage, block, not specified below | Protest by blocking entry and/or exit into building or area, not otherwise specified. |
| 1441 | Obstruct passage to demand leadership change | Obstruct passage, block entry/exit to demand leadership change |
| 1442 | Obstruct passage to demand policy change | Obstruct passage, block entry/exit to demand policy reform |
| 1443 | Obstruct passage to demand rights | Obstruct passage, block entry/exit to demand political, social, economic, or other rights |
| 1444 | Obstruct passage to demand change in institutions, regime | Obstruct passage, block entry/exit to demand major institutional, constitutional, or regime change |
| 151 | Increase police alert status | Need new description. |
| 153 | Mobilize or increase police power | Increase the number of military personnel and/or weapons |
| 155 | Mobilize or increase cyber-forces | Increase the capacity to wage cyber-warfare. |
| 1621 | Reduce or stop economic assistance | Decrease or terminate provision of economic aid |
| 1623 | Reduce or stop humanitarian assistance | Decrease or terminate provision of humanitarian aid |
| 1721 | Impose restrictions on political freedoms | Violate or impose limitations on fundamental political rights such as freedoms of speech, expression, and assembly |
| 176 | Attack cybernetically | Illegal or unauthorized attack on computers, networks, or accounts |
| 180 | Use unconventional violence, not specified below | Use of unconventional forms of violence which do not require high levels of organization or conventional weaponry, not otherwise specified |
| 181 | Abduct, hijack, take hostage | Kidnap, take hostage, hijack, or forcibly seize control of an aircraft, car, bus, ship, etc. |
| 182 | Physically assault, not specified below | Attack physical well-being of individuals without the use of weaponry, not otherwise specified. |
| 1821 | Sexually assault | Sexually abuse, assault sexual integrity of individuals. |
| 1822 | Torture | Torture, inflict extreme pain on individuals. |
| 1823 | Kill by physical assault | Kill individuals by physically assaulting them without the use of weaponry, through beating,torture, lynching, etc. |
| 183 | Conduct suicide, car, or other non-military bombing, not specified  below | The use of explosive devices or improvised explosives outside of military engagements. |
| 1831 | Carry out suicide bombing | Carry out bomb attack with the intention of causing own death as well as other casualties. |
| 1832 | Carry out vehicular bombing | Blow up a car or other vehicle to cause damage to surroundings. |
| 1833 | Carry out roadside bombing | Detonate explosives on the roadside to cause damage and casualties to passers-by. |
| 1834 | Carry out location bombing | The use of pre-placed explosive device(s) with the intent of causing casualties and or/structural damage. |
| 184 | Use as human shield | Use civilians as buffer on the front lines or in other dangerous environments. |
| 201 | Engage in mass expulsion | Force large groups of people or populations out of some territory |
| 203 | Engage in ethnic cleansing | Use mass expulsions and/or mass killings targeting a specific ethnic group. |
| 204 | Use weapons of mass destruction, not specified below | Attack with unconventional weapons that are meant to cause massive destruction and casualties |
| 2041 | Use chemical, biological, or radiological weapons | Attack using chemical, biological, or radiological weapons. |

(Source: http://eventdata.psu.edu/)

Table A2 Pearson & Spearman correlation matrix across NSII variants (*θ_baseline_* , *θ_0.5,_* *θ_0.6,_ θ_pca_* ;exposure = SNA)

| Pearson | *θ_baseline_* | *θ_0.5_* | *θ_0.6_* | *θ_pca_* |
| --- | --- | --- | --- | --- |
| *θ_baseline_* | 1 | 0.993 | 0.993 | 0.994 |
| *θ_0.5_* | 0.993 | 1 | 0.999 | 0.974 |
| *θ_0.6_* | 0.993 | 0.999 | 1 | 0.973 |
| *θ_pca_* | 0.994 | 0.974 | 0.973 | 1 |

| Spearman | *θ_baseline_* | *θ_0.5_* | *θ_0.6_* | *θ_pca_* |
| --- | --- | --- | --- | --- |
| *θ_baseline_* | 1 | 0.995 | 0.995 | 0.997 |
| *θ_0.5_* | 0.995 | 1 | 0.999 | 0.985 |
| *θ_0.6_* | 0.995 | 0.999 | 1 | 0.985 |
| *θ_pca_* | 0.997 | 0.985 | 0.985 | 1 |

Table A3 Threshold sensitivity summary (for 𝜏=0.80,0.90,0.95).

| Year | quantiles | | | Year | quantiles | | |
| --- | --- | --- | --- | --- | --- | --- | --- |
|  | 0.8 | 0.9 | 0.95 |  | 0.8 | 0.9 | 0.95 |
| 2000 | 0.000 | 0.000 | 0.000 | 2012 | 0.167 | 0.333 | 0.636 |
| 2001 | 0.000 | 0.000 | 0.000 | 2013 | 0.095 | 0.190 | 0.364 |
| 2002 | 0.000 | 0.000 | 0.000 | 2014 | 0.186 | 0.364 | 0.727 |
| 2003 | 0.000 | 0.000 | 0.000 | 2015 | 0.256 | 0.500 | 1.000 |
| 2004 | 0.026 | 0.053 | 0.100 | 2016 | 0.333 | 0.667 | 0.786 |
| 2005 | 0.000 | 0.000 | 0.000 | 2017 | 0.209 | 0.409 | 0.818 |
| 2006 | 0.000 | 0.000 | 0.000 | 2018 | 0.186 | 0.364 | 0.727 |
| 2007 | 0.024 | 0.048 | 0.091 | 2019 | 0.167 | 0.333 | 0.636 |
| 2008 | 0.024 | 0.048 | 0.091 | 2020 | 0.071 | 0.143 | 0.273 |
| 2009 | 0.098 | 0.190 | 0.364 | 2021 | 0.095 | 0.190 | 0.364 |
| 2010 | 0.049 | 0.095 | 0.182 | 2022 | 0.098 | 0.190 | 0.364 |
| 2011 | 0.143 | 0.286 | 0.545 | 2023 | 0.143 | 0.286 | 0.545 |

Table A4 Yearly hotspot counts (mean/median) and Jaccard overlaps (mean/median/min/max) vs the fixed baseline (CEN_total_>10,000 & NSII>200).

| tau | 0.80 | 0.90 | 0.95 |
| --- | --- | --- | --- |
| hotspots_mean | 40.958 | 20.750 | 10.708 |
| hotspots_median | 41.50 | 21.00 | 11.00 |
| jaccard_mean | 0.099 | 0.195 | 0.359 |
| jaccard_median | 0.095 | 0.190 | 0.364 |
| jaccard_min | 0.000 | 0.000 | 0.000 |
| jaccard_max | 0.333 | 0.667 | 1.000 |
| years_count | 24 | 24 | 24 |

Table A5 Global Moran’s I Values and Significance Levels for Event Frequency (CEN) and Impact Index (NSII), 2000–2023

| Year | NSII Moran’s I | CEN Moran’s I | Year | NSII Moran’s I | CEN Moran’s I |
| --- | --- | --- | --- | --- | --- |
| 2000 | 0.06 | 0.06 | 2012 | 0.09** | 0.12*** |
| 2001 | 0.08 | 0.07 | 2013 | 0.06* | 0.12*** |
| 2002 | 0.07 | 0.06 | 2014 | 0.08* | 0.10** |
| 2003 | 0.09^**^ | 0.05 | 2015 | 0.23*** | 0.12*** |
| 2004 | 0.08^**^ | 0.04 | 2016 | 0.20*** | 0.11** |
| 2005 | 0.07^**^ | 0.06^*^ | 2017 | 0.16*** | 0.09** |
| 2006 | 0.05 | 0.08^**^ | 2018 | 0.20*** | 0.09** |
| 2007 | 0.05 | 0.07^*^ | 2019 | 0.13*** | 0.08* |
| 2008 | 0.06 | 0.08^*^ | 2020 | 0.16*** | 0.07 |
| 2009 | 0.06 | 0.08^*^ | 2021 | 0.19*** | 0.06 |
| 2010 | 0.08^*^ | 0.09^**^ | 2022 | 0.17*** | 0.02 |
| 2011 | 0.08^*^ | 0.12^***^ | 2023 | 0.18*** | 0.02 |

Note: Global Moran’s I was calculated based on an inverse-distance spatial weights matrix. The matrix was row-standardized. The statistical significance of each index was tested using a random permutation test with 999 permutations to generate pseudo p-values. Significance levels are denoted as follows: p < 0.1 (*), p < 0.05 (**), and p < 0.01(***).
